# Trousseau's Syndrome: A Paraneoplastic Complication.

**DOI:** 10.7759/cureus.66969

**Published:** 2024-08-15

**Authors:** Bryan A Morales Eslava, Joaquín Becerra Bello

**Affiliations:** 1 General Surgery, Hospital Regional PEMEX Villahermosa, Villahermosa, MEX; 2 Vascular Surgery, Angio House, Villahermosa, MEX

**Keywords:** trousseau’s syndrome, dvt (deep vein thrombosis), deep vein thrombosis management, venous thrombosis (ijv), venous thrombophlebitis, cancer-associated thrombosis, deep vein thrombosis (dvt), paraneoplastic syndromes, trousseau's

## Abstract

Trousseau syndrome, also known as thrombophlebitis migrans or migratory superficial thrombophlebitis, is a rare but significant paraneoplastic manifestation associated with various cancers. This syndrome is characterized by the occurrence of recurrent deep or superficial venous thrombosis in patients with malignancies. Patients with cancer have a greatly increased risk of venous thrombosis, especially in the first few months after diagnosis and in the presence of distant metastases. This article describes the case of a 72-year-old female patient who suffered a deep vein thrombosis in the right lower limb, which led to Trousseau syndrome secondary to non-Hodgkin's lymphoma.

## Introduction

Trousseau's syndrome, also known as thrombophlebitis migrans or migratory superficial thrombophlebitis, is a rare but significant paraneoplastic manifestation associated with various cancers. This syndrome was first described by Armand Trousseau in 1865 and is characterized by recurrent deep or superficial venous thrombosis in patients with malignancies [1]. Patients with cancer have a significantly increased risk of venous thrombosis, particularly in the first few months after diagnosis and in the presence of distant metastases. Recent studies have shown that in patients with deep vein thrombosis or pulmonary embolism, malignancy is present in 4 to 20 cases [2]. Prevention of this condition requires timely intervention by the multidisciplinary team caring for the patient, and in the event of a thromboembolic event, early diagnosis and treatment are required to reduce the morbidity and mortality associated with this condition.

This article describes the case of a 72-year-old female patient who suffered a deep venous thrombosis of the right lower limb, leading to Trousseau syndrome secondary to non-Hodgkin's lymphoma. The clinical, pathophysiologic, and treatment aspects of this disease are reviewed.

## Case presentation

**Figure 1:**
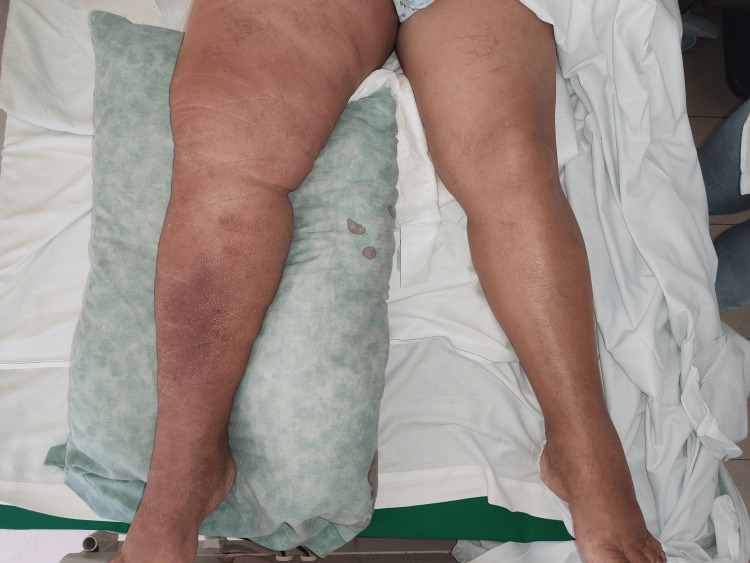
Right lower extremity showing swelling and erythema in comparison with the unnaffected left lower extremity.

A 72-year-old female patient came to the emergency department because she had had an increased right lower extremity volume for a week, accompanied by pain at rest and on mobilization, erythema, and hyperthermia. Physical examination revealed multiple right inguinal nodules 3-6 centimeters in diameter that were non-mobile and not causing pain. The lower extremity showed edema, superficial and deep palpation pain, and preserved but diminished pulses (Figure 1).

**Figure 2:**
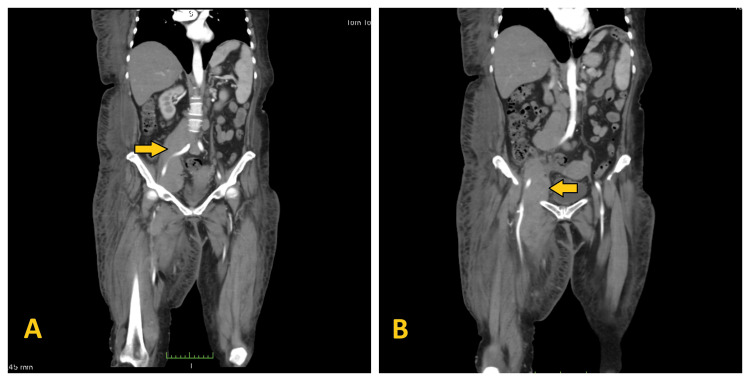
A: Tumor involving right external iliac vein and artery (pointed with arrow). B: Tumor involving right femoral vein and artery (pointed with arrow).

**Figure 3:**
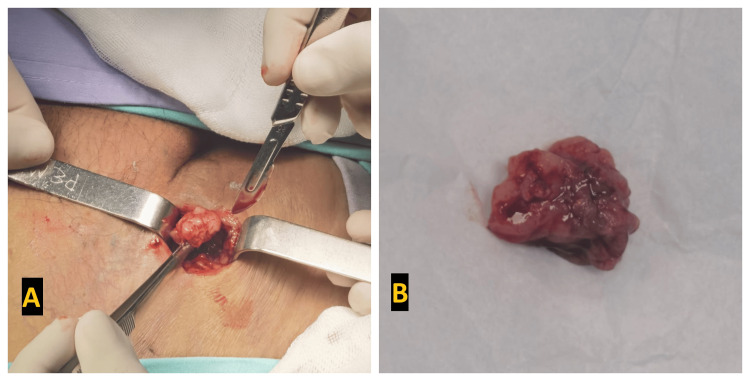
A: Open biopsy of the inguinal lymph node. B: Lymph node specimen obtained from open biopsy.

As part of the diagnostic workup, a Doppler ultrasound of the right groin and right lower extremity confirmed deep vein thrombosis in the right femoral vein, which merged into the common iliac vein. In addition, the patient underwent CT angiography of the abdomen and pelvis, which revealed the presence of a primary tumor in the lower abdomen and pelvis involving the right external iliac vein and right femoral vein and artery (Figure 2). An open biopsy of the inguinal node was performed (Figure 3), and histopathologic examination revealed infiltration by a high-grade diffuse non-Hodgkin's lymphoma consistent with Burkitt's lymphoma.

Once the diagnosis was confirmed, treatment was started with low molecular weight heparin (LMWH), enoxaparin, at a dose of 1 mg/kg SC every 12 hours, together with warfarin at a dose of 5 mg PO every 24 hours. After one week of hospitalization, it was decided to discharge her with oral warfarin as the only treatment, and she was sent to medical oncology to continue her treatment. Two months after discharge, the patient was admitted to the hospital for respiratory distress, where she required intensive care with invasive mechanical ventilation. She was diagnosed with pulmonary thromboembolism and died three days after this admission.

## Discussion

There are several risk factors for Trousseau syndrome, which can be summarized in three categories: patient-related factors, treatment-related factors, and cancer-related factors [3, 4]. Patient-related factors include older age, prolonged immobilization, a personal history of thrombosis, obesity, elevated white blood cell and platelet counts, the presence of an acute infection, and comorbidities such as hypertension and heart disease. As far as treatment-related factors are concerned, the link between chemotherapy drugs and the increased risk of thrombosis has been widely described. In the last category, cancer-related factors, we note that cancer cells themselves induce a procoagulant state through the production of clotting factors (mainly tissue factor (TF)), proinflammatory cytokines, and increased expression of thrombin receptors, as well as endothelial activation induced by the direct interaction of tumor cells with the vascular endothelium, triggering the release of proinflammatory and procoagulant molecules [3]. Other authors have described the pathophysiology of Trousseau syndrome and linked it to Virchow's triad, which consists of stagnant or turbulent blood flow, changes in the vessel walls, and a hypercoagulable state [4].

In the clinical picture, Trousseau syndrome typically presents with recurrent deep vein thrombosis, which can manifest as follows [4, 5]:

Migratory Thrombophlebitis: characterized by the successive appearance of painful, swollen, erythematous areas on the extremities.

Pulmonary Thromboembolism: may result from the spread of thrombi from superficial or deep veins into the pulmonary circulation.

Arterial Thromboembolic Events: Less common but possible, with a risk of cerebral or peripheral ischemic events.

The diagnosis of Trousseau syndrome is based on clinical history, physical examination, and diagnostic tests, including venous Doppler to detect deep vein thrombosis, computed tomography or magnetic resonance imaging to assess the extent and possible origin of the thrombi, and tumor markers to identify the underlying neoplasm.

The treatment of Trousseau syndrome aims to reduce mortality and morbidity and improve the patient's quality of life. This includes treating the symptoms as well as the underlying thrombosis and cancer. According to the National Comprehensive Cancer Network (NCCN) guidelines for cancer-associated venous thromboembolism, version 2.2021, there are three alternatives for the initiation of anticoagulation therapy in this patient group, namely direct oral anticoagulants (DOACs), low molecular weight heparins (LMWHs; enoxaparin, dalteparin) and warfarin. The duration of the chosen treatment depends on whether the thrombosis is related to the presence of a catheter, in which case a duration of 3 months is recommended, or whether the thrombosis is not related to the presence of a catheter, in which case, it is recommended that the duration of treatment be indefinite and as long as the cancer is active, being treated, or there are risk factors for recurrence [6]. Specific treatment of the primary tumor may include surgery, chemotherapy, radiotherapy, or biological therapies.

The prognosis of Trousseau syndrome depends mainly on the stage and type of the underlying cancer and the response to treatment. Mortality is influenced by the presence of pulmonary thromboembolism and the ability to control the neoplastic disease [4, 5].

## Conclusions

Trousseau syndrome is a serious but treatable complication associated with advanced cancer. A multidisciplinary approach involving oncologists, hematologists, and vascular surgeons is essential for effective management and improved clinical outcomes. Understanding the underlying pathophysiologic mechanisms is crucial for early diagnosis and implementation of appropriate therapeutic strategies to mitigate the risks associated with this paraneoplastic disease.

This case underscores the importance of monitoring for thrombotic complications in cancer patients and highlights the need for timely diagnosis and treatment to optimize clinical outcomes.

## References

[1] Metharom P, Falasca M, Berndt MC (2019). The history of Armand Trousseau and cancer-associated thrombosis. Cancers (Basel).

[2] Blom JW, Doggen CJ, Osanto S, Rosendaal FR (2005). Malignancies, prothrombotic mutations, and the risk of venous thrombosis. JAMA.

[3] Varki A (2007). Trousseau's syndrome: multiple definitions and multiple mechanisms. Blood.

[4] Ikushima S, Ono R, Fukuda K, Sakayori M, Awano N, Kondo K (2016). Trousseau's syndrome: cancer-associated thrombosis. Jpn J Clin Oncol.

[5] Dicke C, Langer F (2015). Pathophysiology of Trousseau's syndrome. Hamostaseologie.

[6] Streiff MB, Holmstrom B, Angelini D (2021). Cancer-Associated Venous Thromboembolic Disease, Version 2.2021, NCCN Clinical Practice Guidelines in Oncology. J Natl Compr Canc Netw.

